# Evaluation of Phenolic Compounds and Antioxidant Activity in Three Black Cherry Tomato Varieties Grown Under Greenhouse Conditions

**DOI:** 10.3390/plants14081173

**Published:** 2025-04-10

**Authors:** Josué Daniel Hernández-Vega, Ixchel Parola-Contreras, Erik Gustavo Tovar-Pérez, Ramón Gerardo Guevara-González, Humberto Aguirre-Becerra, Ana Angélica Feregrino-Pérez, Luis Miguel Contreras-Medina, Rosario Guzmán-Cruz

**Affiliations:** 1 Centro de Investigación Aplicada en Biosistemas (CIAB), Facultad de Ingeniería, Universidad Autónoma de Querétaro, Campus Amazcala, Carr. Chichimequillas-Amazcala Km 1 S/N, Amazcala, El Marques 76265, Querétaro, Mexico; jhernandez44@alumnos.uaq.mx (J.D.H.-V.); ramon.guevara@uaq.mx (R.G.G.-G.); humberto.aguirreb@uaq.mx (H.A.-B.); miguel.contreras@uaq.mx (L.M.C.-M.); 2 TecNM/Tecnológico de Estudios Superiores de Chimalhuacán, Ingeniería Industrial, Ingeniería de Procesos Sustentables ITESCHIM-CA-02, Chimalhuacán 56335, Estado de México, Mexico; ixchelparola@teschi.edu.mx; 3 Facultad de Ingeniería, Universidad Autónoma de Querétaro, Campus Amealco, Camacho Guzmán, Fracc. Rinconada de Bonfil, Amealco 76850, Querétaro, Mexico; erik.tovar@uaq.mx; 4 C.A. Bioingeniería Básica y Aplicada, Facultad de Ingeniería, Universidad Autónoma de Querétaro, Campus Amazcala, Carr. Chichimequillas-Amazcala Km 1 S/N, Amazcala, El Marques 76265, Querétaro, Mexico; geli@uaq.mx

**Keywords:** antioxidants, polyphenols, flavonoids, anthocyanins, carotene, enzymatic activity

## Abstract

Given the importance of phenolic compounds and antioxidant capacity in plant defense and human health, this study aimed to evaluate black cherry tomatoes’ polyphenol, flavonoid, anthocyanin, and carotenoid content and enzymatic activity under greenhouse conditions. Black cherry tomato varieties—Indigo Cherry Drops, Indigo Rose, and Kumato—were cultivated from seed to the third harvest. Total polyphenols, flavonoids, anthocyanins, β-carotenoids, antioxidant capacity 2,2-diphenyl-1-picrylhydrazyl (DPPH), 2,2’-azino-bis (3-ethylbenzothiazoline-6-sulfonic acid) (ABTS), and enzymatic activities, including superoxide dismutase (SOD), catalase (CAT), proline (PRO), and superoxide dismutase (PAL), were measured and compared. The Kumato variety exhibited significantly higher total polyphenols, flavonoids, and carotenoids, along with enhanced antioxidant activity (DPPH, ABTS) and enzymatic activity (CAT, PAL) compared to Indigo Rose and Indigo Cherry Drops, with free radical inhibition of 87.0% (DPPH) and 74.72% (ABTS). SOD activity was highest in Indigo Rose (0.21 U/mg protein), while proline levels were significantly higher in Kumato and Indigo Cherry Drops (6.40 and 6.63 U/mg protein). These findings highlight the antioxidant potential of black cherry tomatoes and their high potential nutritional value for consumers. Future research should explore how environmental factors influence their biochemical composition and potential applications in functional food.

## 1. Introduction

Free radicals are chemical molecules with one or more loosed electrons in their structure. These compounds can cause severe damage to living beings despite their short lifetime, as they can interfere with different biochemical paths [1]. Additionally, these radicals have high reactivity, promoting the production of more free radicals through many chemical reactions associated with the same molecule or adjacent molecules [2]. In plants, these radicals come from reactive oxygen species (ROS), which include singlet oxygen (^1^O_2_), hydroxyl radical (HO^−^), superoxide anion radical (O_2_^−^), and hydrogen peroxide (H_2_O_2_) [3]. These ROS play a critical role in vegetative growth and flowering by regulating redox levels, stress signaling, cell differentiation, and biochemical reactions. Moreover, they can also induce cell differentiation and programmed cell death [4].

Plants have developed physiological and metabolic responses to counteract oxidative stress from harsh environmental conditions (e.g., drought, salinity, extreme temperatures, poor nutrition, pests, and pathogens). These responses include stimulating stress-sensitive genes and activating enzymatic and non-enzymatic antioxidant systems that help mitigate the harmful effects of ROS [5]. Additionally, adaptation is crucial for plant survival in chronic biotic and abiotic stress conditions. Some mechanisms include photosynthesis adjustment, hormonal responses, accumulation of secondary metabolites, physiological adaptations, and genetic regulation of stress responses [6]. Key enzymatic activity, such as superoxide dismutase (SOD) and catalase (CAT), is considered the first line of defense that minimizes ROS toxicity in a preventive action mechanism [5,7]. Additionally, non-enzymatic defense mechanisms involve compounds like proline (PRO) that stabilize the protein structures and buffer cellular redox state and cytosolic pH when plants face stress [8]. The phenylpropanoid pathway, driven by the enzyme phenylalanine ammonia-lyase (PAL), is another crucial antioxidant system that produces secondary metabolites, such as anthocyanins and flavonoids, that are a second line of defense against the ROS toxic accumulation [5,9].

Secondary metabolites are widely studied non-enzymatic ROS scavengers with significant antioxidant properties [4]. The type and amount of these biochemical compounds depend on the plant type [10]. In tomatoes, biochemical compounds, such as polyphenols, flavonoids, and carotenoids, are the primary antioxidant compounds contributing to plant defense, a standard for fruit quality that influences market value and maintains tomato homeostasis [11,12]. In parallel, phenolic compounds can modulate the expression of genes related to antioxidant enzymes, establishing a synergistic relationship between both systems [13,14]. ROS-inhibiting activity continues when humans consume the fruit, affecting human health and providing nutritional benefits [5]. Plants coordinate their enzymatic and non-enzymatic mechanisms to achieve antioxidant activity. On one hand, enzymes, such as SOD, CAT, and PAL, neutralize excessive ROS. On the other hand, phenolic compounds reinforce the defense against ROS by scavenging free radicals and modulating gene expression [15].

Cherry tomatoes, particularly black cherry tomatoes, exhibit a unique biochemical profile due to their diverse phenolic composition. The pigmentation of black cherry tomatoes is influenced by anthocyanins, like those found in blueberries and bilberries, which protect plants from UV radiation and have applications in food and medicine [16]. Carotenoids and anthocyanins provide distinctive coloration and enhance black cherry tomatoes’ nutritional and antioxidant properties [17].

Those tomatoes represent an interesting model for studying the pathways of these compounds as they interact with endogenous antioxidant enzymes to mitigate oxidative stress. However, the specific interactions between anthocyanins and antioxidant enzymes in these varieties remain poorly explored, offering an opportunity to investigate how these components collaborate to protect the plant from oxidative stress [15].

Tomatoes are one of the three main vegetables grown worldwide, producing 180 to 190 million tons in 2023 [18]. Mexico ranks among the top ten producers, yielding 4 to 5 million tons annually [19]. The production area of this fruit consisted of 48,042 ha, where 5% were grape and cherry tomatoes in 2022. In the same year, 50,000 tons of cherry tomatoes were harvested at USD 25.00 per box [20]. The biggest problems with the tomato crop are environmental variations, such as geographic conditions, plague presence, seasonal influence on crop cycles, and low handwork capacity. Adopting advanced agricultural techniques and crop protection under greenhouses has increased the yield of this product in Mexico in the last decade. Environmental variations and water availability for tomato crops are challenges that increase production costs, overexploitation of aquifers, low yields, and inequity for small producers [21].

Although black cherry tomato crop management is the same as that of other cherry tomatoes [22], the morphological behavior of black cherry tomato plants is analogous to conventional cherry tomato plants [23]. However, there is limited research on black cherry tomato production, biochemical composition, and economic potential, as studies primarily focus on conventional varieties, leaving a gap in knowledge regarding this variety’s phenolic content, antioxidant capacity, and enzymatic activity. This study aims to quantify and compare the total content of polyphenols, flavonoids, anthocyanins, and carotenoids and evaluate the antioxidant capacity and enzymatic activities of three varieties of black cherry tomato: Indigo Rose, Kumato, and Indigo Cherry Drops. Tomatoes were grown in greenhouses under stress-free conditions and provide valuable insight into the relationship between fruit pigmentation and free radical scavenging properties. The findings enhance our understanding of the free radical scavenging capacity of black cherry tomatoes, highlighting their potential to improve fruit quality and market value while informing producers and consumers about their health benefits. In addition, it will increase knowledge about ROS protections of emerging tomato cultivars.

## 2. Results

### 2.1. Biological Material

**Figure 1:**
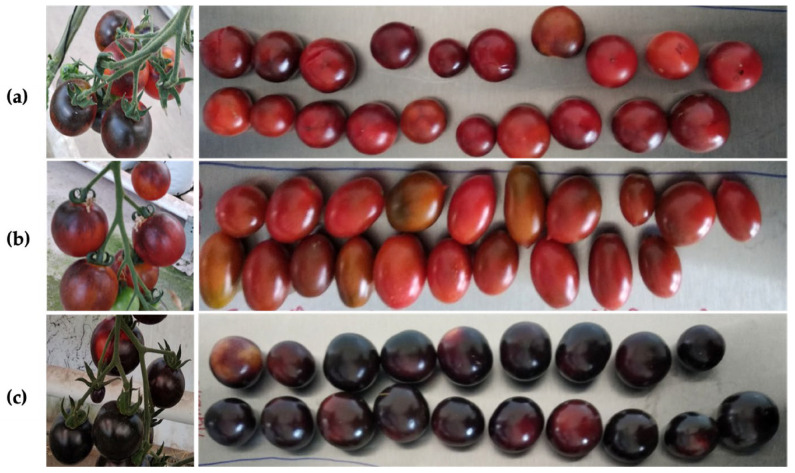
Tomato cluster of the (**a**) Indigo Rose, (**b**) Indigo Cherry Drops, and (**c**) Kumato varieties.

Black cherry tomato varieties (Indigo Rose, Kumato, and Indigo Cherry Drops) were cultivated and harvested. Plant height, number of clusters/plants, growth, and flowering rates presented no difference. The Kumato variety had the tallest plants, and the Indigo Cherry Drops variety had the highest number of clusters by plant. Figure 1 shows a bunch of each tomato variety.

### 2.2. Enzymatic Activity

**Figure 2:**
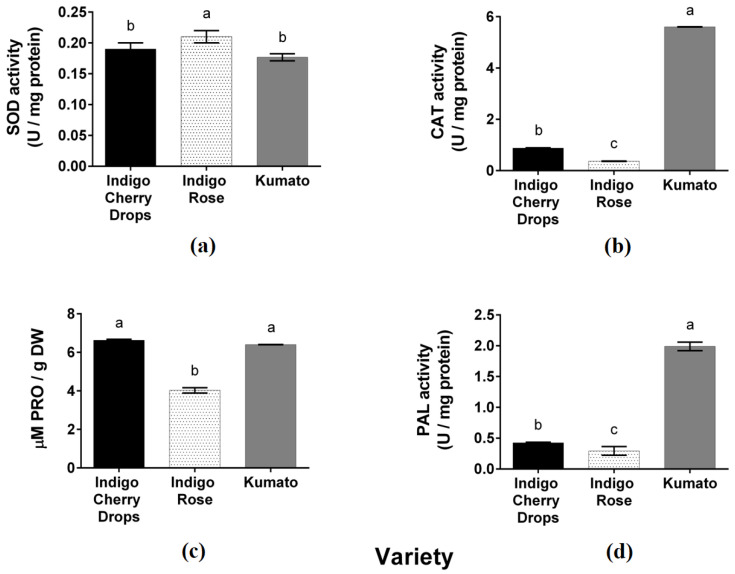
(**a**) SOD, (**b**) CAT, (**c**) PRO, and (**d**) PAL activity (U/mg protein) of three black cherry tomato varieties. Different letters indicate significant differences according to ANOVA and Tukey’s test (α = 0.05).

The CAT, SOD, PRO, and PAL enzymatic activities of black cherry tomatoes are shown in Figure 2.

SOD activity in the Indigo Rose variety presented a value of 0.21 U/mg protein (Figure 2a) and fold ratios of 1.19 and 1.09 compared to the Kumato and Indigo Cherry Drops varieties, respectively. Subsequently, CAT activity in the Kumato variety was 5.60 U/mg protein (Figure 2b), corresponding to fold ratios of 0.96 and 1.59 compared to the Indigo Cherry Drops and Indigo Rose varieties, respectively. The Kumato variety exhibited higher levels of stress-responsive enzymes, triggering the phenylpropanoid pathway. PRO enzymatic activity was 6.63 μM PRO/g DW (Figure 2c) in the Indigo Cherry Drops variety, followed by the Kumato variety, with a 1.03-fold increase, and the Indigo Rose variety, with a 1.64-fold increase. Finally, PAL quantification in the Kumato variety was 1.99 U/g protein (Figure 2d) with fold ratios of 4.69 and 6.79 compared to the Indigo Cherry Drops and Indigo Rose varieties, respectively.

### 2.3. Phenolic Compounds

**Figure 3:**
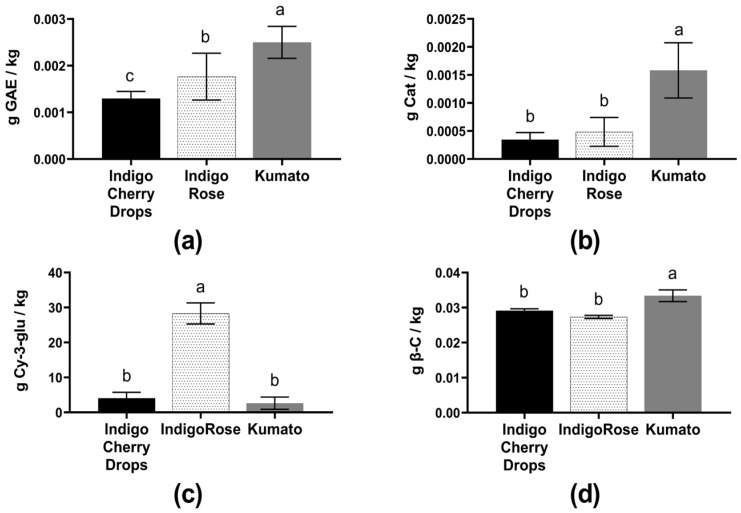
(**a**) Total polyphenol content, (**b**) flavonoid content, (**c**) total anthocyanin content, and (**d**) β-carotene content in the three black cherry tomato varieties. Different letters indicate significant differences using ANOVA and Tukey’s test (α = 0.05).

Phenolic compounds are secondary metabolites for plant defense against ROS produced due to ultraviolet radiation and pathogen attacks. These phytochemicals also participate in photosynthetic processes and contribute to the sensorial characteristics of fruits (e.g., color, astringency, taste, and aroma) [6]. Figure 3a shows the total polyphenol content in the Indigo Cherry Drops, Indigo Rose, and Kumato varieties. The Kumato variety exhibited a 1.38-fold and 1.82-fold increase compared to the Indigo Rose and Indigo Cherry Drops varieties, respectively.

As shown in Figure 3b, the Kumato variety exhibited fold ratios of 5.15 and 4.65 compared to Indigo Rose and Indigo Cherry Drops, respectively. The Kumato variety demonstrated a more vigorous response, making its antioxidant properties potentially highly beneficial for human health.

Regarding anthocyanin content, the Indigo Rose variety had fold ratios of 6.95 compared to Indigo Cherry Drops and 10.75 compared to the Kumato variety (Figure 3c). Additionally, the Kumato variety exhibited a significant difference in β-carotene content (Figure 3d), while the other two varieties had fold ratios of 1.14 and 1.22 relative to Indigo Cherry Drops and Indigo Rose, respectively.

### 2.4. Interaction Between Endogenous Antioxidant Enzymes and Antioxidant Compounds

**Figure 4:**
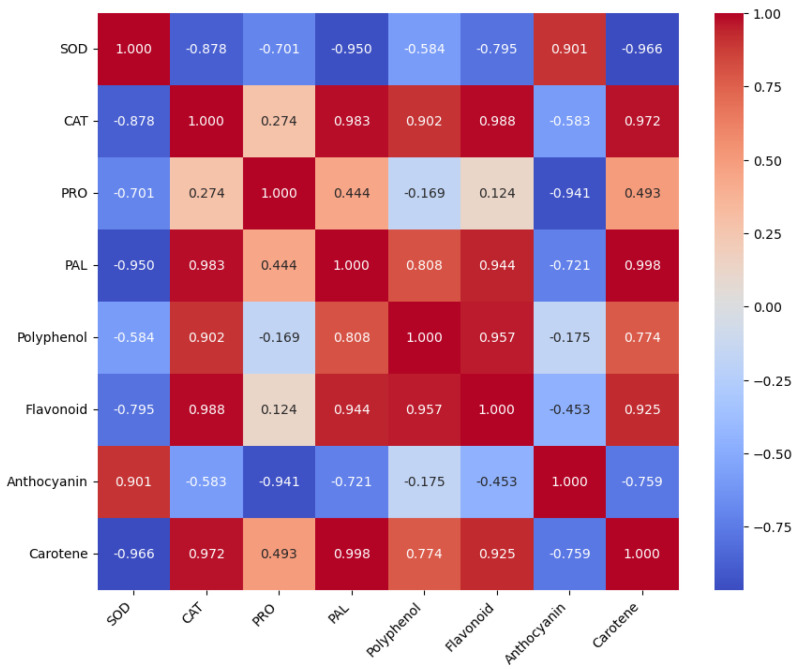
Heat map of the correlation matrix of enzymatic activities and non-enzymatic compounds.

To analyze the interaction between enzymatic activities and non-enzymatic compounds, we performed a correlation analysis to evaluate the relationships between SOD, CAT, PRO, PAL, total phenolic acids, total flavonoids, anthocyanins, and β-carotenes. We used Pearson’s correlation coefficient to measure the strength and direction of the linear relationships between variables (Figure 4).

As shown in Figure 4, there are strong positive correlations between CAT and flavonoids (0.988), CAT and carotene (0.972), and CAT and polyphenol (0.902). This indicates that higher catalase activity also increases the content of these antioxidant compounds.

In addition, there are strong positive correlations between PAL and carotene (0.998) and PAL and flavonoids (0.944). This suggests a direct relationship between PAL activity (key in synthesizing phenolic compounds) and flavonoid and carotenoid levels.

Similarly, polyphenol and flavonoids (0.957) are found. This is expected, as flavonoids are a type of polyphenol. Finally, flavonoids and carotene (0.925) are found. These antioxidant compounds tend to be present together in varieties with high levels of antioxidant defense.

On the other hand, there are strong negative correlations between SOD and PAL (−0.950) and SOD and carotene (−0.966). This could indicate a trade-off between enzymatic and non-enzymatic defense. That is, varieties with higher SOD activity may require less carotenoids or PAL activity.

SOD and CAT (−0.878) also show strong negative correlations. Although both are antioxidant enzymes, their action may be inversely regulated in certain varieties.

Anthocyanins correlate negatively with almost all other enzymes (CAT, PRO, PAL, polyphenol, flavonoids, and carotene). This suggests that anthocyanins may follow a different metabolic pathway or compete for metabolic resources.

### 2.5. Antioxidant Activity

Table 1 shows the 2,2’-azino-bis (3-ethylbenzothiazoline-6-sulfonic acid (ABTS) and 2,2-diphenyl-1-picrylhydrazyl (DPPH) radical scavenging determined in this study. In addition, Table 1 presents the percentage rate of inhibition of Trolox.

The TEAC for the ABTS assay of the Kumato variety was 1.34-fold compared to the Indigo Rose variety and 1.93-fold compared to the Indigo Cherry Drops variety.

## 3. Discussion

Enzymatic activities involved in the stress response play a crucial role in mitigating oxidative stress, mainly through enzymes like SOD, CAT, and PRO. Additionally, other enzymes activate the biosynthetic pathways of specialized metabolites, such as PAL, which serves as a precursor enzyme in the phenylpropanoid pathway [24].

Maintaining a balance between antioxidant and pro-oxidant molecules is essential throughout plant growth, development, and harvest. Any disruption in this balance can lead to oxidative stress, triggering the biosynthesis of specialized molecules to restore equilibrium. The activities of antioxidant defense enzymes (SOD, CAT, PRO) and phenol pathway-related enzymes (PAL) serve as key indicators of plant immune response, functioning as signaling molecules [25].

SOD is the first line of defense against oxidative stress, catalyzing the conversion of superoxide radicals into H_2_O_2_ and O_2_, preventing the formation of free radicals, promoting cellular health, and reducing disease susceptibility [26]. Previous studies have reported varying SOD enzymatic activity levels, including 3538.5 U/mg protein [27], 100–166.84 U/mg protein in Micro-Tom and Red Rain tomato leaves [28], 68.68–72.72 U/mg protein in five different tomato varieties [29], and 65.65 U/mg protein in the Huno F1 variety [30].

Furthermore, [31] obtained 4.53 U/mg protein in the Huno F1 fruit variety. CAT enzymes, vital for ROS detoxification, are primarily localized in glyoxysomes, peroxisomes, and, to a lesser extent, mitochondria [32]. This study recorded higher CAT activity than previously reported values, ranging between 0.1 and 5.3 U/mg protein [27,28,29], with some exceptions, such as tomato seeds with 24.29 U/mg protein [33] and the Huno F1 fruit variety, where CAT activity ranged from 268.8 to 618 U/mg protein [29,30].

PRO acts as an osmolyte, storing carbon and nitrogen while regulating cellular redox potential. It is a key component of the antioxidant defense system, stabilizing subcellular structures and macromolecules, serving as part of the signal transduction pathways, and regulating stress-sensitive genes. PRO accumulation occurs during osmotic stress and plays a crucial role in germination [34]. However, limited research has been conducted on PRO enzymatic activity in tomato fruit. Previous studies reported activity levels of 0.0038 and 0.0035 μM PRO/g DW in Micro-Tom and Red Rain leaves, respectively [28].

The PAL enzyme catalyzes reactions that produce protective compounds, including phenols, flavonoids, anthocyanins, coumarins, and lignins. Its activity is stimulated by environmental factors, such as low temperatures and light exposure [8]. This study found that the Kumato variety exhibited greater PAL activity than the Huno F1 fruit [30]. However, the Indigo Cherry Drops and Indigo Rose varieties displayed lower enzymatic activity than the Huno F1 variety. Previous studies on the Qianxi fruit variety reported PAL activity levels of 161.91 and 103.16 U/mg protein [35,36]. The high PAL activity observed in the Kumato variety is directly associated with increased production of phenolic compounds, reinforcing its role in plant defense and stress tolerance.

This study compares three black cherry tomato cultivars, evaluating their phytochemical content and antioxidant capacities. The findings highlight the metabolic responses of tomato plants to abiotic stressors. The observed variability in enzymatic activity among different tomato varieties suggests a genetic basis for stress tolerance. Similar methodologies can be applied to other plant species rich in polyphenols and carotenoids to explore their antioxidant potential.

Furthermore, the results indicate that CAT is a positive indicator of non-enzymatic antioxidant content, particularly flavonoids and carotenoids. This implies that varieties with high catalase activity possess more comprehensive antioxidant mechanisms. PAL, which is involved in phenol biosynthesis, correlates well with flavonoids and carotenoids, reinforcing its role as a metabolic pathway for these compounds.

The negative correlations between SOD and several non-enzymatic compounds may suggest a compensatory mechanism; when antioxidant enzymes are more active, non-enzymatic chemical support may be less necessary, and vice versa. In contrast, anthocyanin displays behavior that is dissociated from the others. Its concentration is notably high in certain specific varieties, such as Indigo Rose, yet it does not correlate with the other antioxidants. This indicates a distinct defense profile or specific coloration.

This knowledge has significant implications for agricultural breeding programs to develop stress-resistant cultivars with enhanced antioxidant properties [37]. Additionally, the distinct phytochemical profiles of these black cherry tomatoes may influence consumer preferences and market value, highlighting their potential role in producing high-value, health-promoting crops and developing functional foods or dietary supplements in the nutraceutical industry.

Phenolic compounds are secondary metabolites involved in plant defense mechanisms against ROS, playing a role in photosynthesis and contributing to fruit sensory attributes, such as color, taste, and aroma [6]. Previous studies have reported total polyphenol concentrations of 0.46, 5.65, and 6.78 g GAE/kg DW in Kumato, Red Cherry, and Yellow Cherry varieties, respectively [38]. On the other hand, another study found that the black cherry tomato variety ’Black Q’ exhibited high levels of chlorophylls, β-carotene, total flavonoids, and anthocyanins. In contrast, the red variety ‘Snacktom’ stood out only for its high lycopene content [10]. Kumato has also been reported to contain 33.57 g GAE/kg DW [39], while the Huno F1 variety exhibited 30.86 g GAE/kg DW [30]. Comparatively, the Sun Black variety had a polyphenol concentration of 8.56 g GAE/kg DW [40]. In the TY-605 variety, the highest phenolic concentration recorded was 2.902 g GAE/kg DW [16].

Furthermore, in [41], 0.44, 0.46, and 0.32 g GAE/kg DW was determined in the Red Cherry, Brown Cherry, and Brown Beefsteak varieties, respectively. In comparison, the Qianxi variety reached 0.24 g GAE/kg DW [36]. Phenols contribute to the structural integrity of plant cell walls and protect against radiation, making them key compounds in plant defense. The higher phenolic content observed in the Kumato variety supports its classification as a nutraceutical food with potential health benefits [42,43]. In [11], the variety had 1.13 g Naringin Eq/kg DW. Furthermore, flavonoids, known for their antioxidant properties and role as iron chelators, have been measured in various tomato varieties, with the highest concentrations reported in the Sun Black (1.11 g Cat Eq/kg DW) [40] and Huno F1 (1.09 g Cat Eq/kg DW) varieties [38]. Furthermore, the Qianxy variety reached 0.37 g Cat Eq/kg DW [36]. Furthermore, [38] found 0.35, 0.31, and 0.25 g Cat Eq/kg DW in the Yellow Cherry, Kumato, and Red Cherry varieties, respectively.

Finally, the study by [16] of the Oleh TY variety found enhanced 0.02 g CAT Eq/kg DW. Their yellow color characterizes flavonoids, iron chelators that are considered excellent antioxidants due to their chemical properties. The Kumato variety showed a stronger reaction, making its antioxidant properties advantageous for health, contributing positively to human health by combating oxidative stress and related diseases [44].

Anthocyanins, primarily responsible for UV protection in fruits with blue pigmentation, are present in black cherry tomatoes, such as Indigo Rose. Previous studies have reported anthocyanin levels of 17 and 0.1 g Cy-3-gly/kg DW in the peel and pulp, respectively [45]. Other studies have recorded values of 3.99 g Cy-3-gly/kg DW in Indigo Rose seeds [46], 1.16 g Cy-3-gly/kg DW in the Sun Black variety [40], and lower concentrations in Yellow Cherry, Red Cherry, and Kumato [38]. Ref. [40] obtained the highest carotene accumulation reported, with 0.11 g β-Car/kg DW in the Sun Black variety. Meanwhile, ref. [38] found 0.10, 0.03, and 0.001 g β-Car/kg DW in the Red Cherry, Kumato, and Yellow Cherry varieties. For the Kumato variety, [38] reported 0.066 g β-Car/kg DW. The highest variety of β-Carotene in [16] was Rubyking, with 0.036 g β-Car/kg DW.

Carotenoids, derived from tetraterpenes, are responsible for the red color of tomatoes and protection from UV radiation. In the Kumato variety, terpenes are key in light energy absorption, providing photoprotection and contributing to vitamin A synthesis [47]. These mechanisms benefit the plant and may support human health by preventing cancer, modulating the immune system, regulating the cell cycle, and regulating hormonal signaling. Due to its higher antioxidant activity and phenolic compound concentration, Kumato stands out as a nutraceutical option with potential benefits in combating oxidative stress and related diseases [47].

The Kumato variety exhibited the highest ABTS antioxidant activity (378.17 mmol T/kg DW) [11], followed by TY-605 (61.17 mmol T/kg DW) [16], Sun Black (31.64 mmol T/kg DW) [40], and Huno F1 (13.19 mmol T/kg DW) [30]. The Indigo Rose variety presented different values in the peel (6.15), pulp (1.32), and seed (0.40 mmol T/kg DW) [17].

In the DPPH assay, Kumato also showed significant antioxidant capacity. Indigo Cherry Drops had 58.70% inhibition, while Indigo Rose reached 41.38%. The inhibition rate in this study was higher in the ABTS assay compared to previous reports, such as [16,17,30], which ranged from 0.04% to 61.17%. Indigo Rose reached 65.83% inhibition in the ABTS assay. Other reported values include Red Cherry (26.06 mmol T/kg DW), Brown Cherry (0.27 mmol T/kg DW), and Brown Beefsteak (0.16 mmol T/kg DW) [41]. The highest value for TY-605 was recorded at 21.74 mmol T/kg DW [16].

In addition to all of the above, it is important to consider the in vivo function of endogenous antioxidants and plant compounds in human and animal models because redox balance in living organisms is essential for maintaining normal physiological functions. However, factors like aging, exposure to pollutants, an inadequate diet, or chronic diseases can generate excess reactive oxygen species (ROS), which causes oxidative damage at the cellular level. In this context, endogenous and exogenous antioxidants play a key role in protecting against oxidative stress [48].

The body itself synthesizes endogenous antioxidants (SOD, CAT, and PAL) and constitutes the first line of defense against free radicals. These enzymes are especially expressed in tissues with high metabolic activity, such as the liver, brain, and kidneys, and their activity can increase in response to stress conditions, infections, or a diet rich in plant antioxidants [49]. On the other hand, antioxidant plant compounds, such as polyphenols, flavonoids, carotenoids, and anthocyanins, found in fruits, vegetables, and medicinal plants, complement the antioxidant action. Studies in animal models have shown that diets enriched with plant compounds, such as anthocyanins, reduce oxidative stress markers, improve memory, and protect against liver damage [50]. In humans, regular consumption of fruits rich in flavonoids and carotenoids is associated with a lower incidence of cardiovascular and neurodegenerative diseases and certain types of cancer [51].

The action of these compounds depends not only on their direct antioxidant capacity but also their bioavailability and synergy with endogenous enzymes. Therefore, endogenous antioxidants and plant compounds form an integrated and complementary defense system against oxidative damage. While endogenous enzymes control the internal redox balance, exogenous antioxidants modulate these responses and offer additional protection. Their study in animal and human models allows us to better understand their therapeutic and preventive potential, especially in chronic diseases, aging, and exposure to environmental pollution.

Future research should elucidate the genetic mechanisms underlying variations in enzymatic activity and phenolic compounds across tomato varieties to enhance breeding programs, plant stress resilience, and fruit antioxidant properties. Additionally, studies should explore the influence of environmental factors, such as temperature and light exposure, on enzyme activity and metabolite production. Further investigations are needed of the health benefits of antioxidant-rich tomato varieties in human diets.

A multidisciplinary approach integrating plant physiology, genetics, and nutrition will provide a comprehensive understanding of the interactions between plant metabolism, environmental stressors, and human health. This knowledge could contribute to better agricultural practices, the establishment of dietary recommendations, and the development of functional foods with enhanced health benefits.

## 4. Materials and Methods

### 4.1. Establishment of the Crop

Black cherry tomato varieties (Indigo Rose, Indigo Cherry Drops, and Kumato) were cultivated under controlled greenhouse conditions to ensure standardized growing conditions and minimize external variability. This approach allowed for precise regulation of environmental factors, such as temperature, humidity, light exposure, irrigation, and nutrient availability, ensuring that observed differences in biochemical composition were attributable to genetic and metabolic factors rather than external stressors.

The study was carried out in a 30 m^2^ chapel-type greenhouse at the Amazcala campus of the Autonomous University of Queretaro, in the municipality of El Marques, at the geographical coordinates 20°42′19.9″ N and 100°15′35.6″ W. The tomato was grown during the spring–summer 2023 cycle.

The growing process for black cherry tomatoes was carried out as reported in [23]. The tomato cherry varieties were marked and placed randomly. The greenhouse had natural ventilation, supplemented with shade nets to prevent excessive UV exposure and to maintain optimal temperature conditions. Temperature was monitored using digital sensors, presenting an average range of 22–32 °C during the day and 16–20 °C at night. Natural air circulation maintained relative humidity within 60–70%. Plants received only natural sunlight, with no artificial lighting supplementation, allowing the experiment to reflect actual greenhouse production conditions. A drip irrigation system with emitters near each plant ensured uniform and precise water delivery, preventing drought and overwatering. After four weeks, seedlings in the trays were irrigated with a Steiner nutrient solution adapted to the phenological stage (Table 2). Six-week-old seedlings were transplanted into the greenhouse. The growth stage was from the 38th to the 59th day after sowing until the first flowers sprouted. The fruit growth stage begins from that point to the end of the crop.

Plants exhibiting signs of stress (e.g., leaf curling, necrosis, and chlorosis) were excluded from the analysis to ensure unbiased results.

### 4.2. Biological Material

Black cherry tomatoes were randomly selected from the third harvest on the 120th day after sowing. Twenty tomatoes of the three varieties were washed, dried, sliced, and dehydrated on a stove at 30 °C for 36 h. Then, the samples were liquefied to reduce them to granular solids and preserve them.

### 4.3. Quality Control Procedures for the Determination of Enzymatic Activity, Phenolic Compounds, and Antioxidant Activity

Various quality control (QC) strategies were implemented for each applied methodology to ensure data accuracy and reliability.

#### 4.3.1. Equipment Calibration

UV-Vis Spectrophotometry. Before each analysis, the spectrophotometer was calibrated using reference blanks and appropriate standards to ensure reading accuracy.

Enzymatic Activity Assays. A microplate reader was pre-calibrated with commercial standards and appropriate buffer solutions to ensure consistent measurements.

#### 4.3.2. Use of Reference Materials and Standards

Certified standard solutions of phenolic compounds (such as gallic acid, catechin, and chlorogenic acid) were used to elaborate calibration curves for total phenolic determination.

For antioxidant activity assays (DPPH, ABTS, and FRAP), Trolox and ascorbic acid standard solutions were used to generate reference curves.

#### 4.3.3. Sample Replication

All determinations were performed in triplicate to minimize experimental errors and ensure result reproducibility.

Internal control samples were processed in each analytical batch to detect potential variations between experimental runs.

#### 4.3.4. Method Validation and Precision Control

The linearity of calibration curves was evaluated using correlation coefficients (R^2^ > 0.99) to ensure their reliability.

Intra- and inter-day precision was determined by analyzing variability in repeated measurements of standards and actual samples.

The recovery of phenolic compounds and antioxidants was assessed by adding known standards to actual samples and calculating the recovery percentage (acceptable within the 95–105% range).

These procedures ensured the accuracy and reliability of data obtained for enzymatic activity, phenolic compound content, and antioxidant activity determination.

### 4.4. Enzymatic Activity Determination

#### 4.4.1. Sample Preparation for Enzymatic Assays

Black cherry tomato samples (0.3 g) were homogenized with 1 mL of cold extraction buffer, blended, and centrifuged at 13,000 rpm for 20 min at 4 °C. The supernatants were used to quantify the enzyme activities through spectrophotometry. The protein concentration in enzymatic extracts was determined using bovine serum albumin as the standard [53] (Sigma Aldrich, St. Louis, MO, USA).

#### 4.4.2. SOD Activity Assay

The SOD activity (EC 1.15.1.1) was analyzed through the inhibition of the photochemical reduction of Nitro Blue Tetrazolium (NBT) [52]. The reaction consists of 1.5 mL of 50 mMol potassium phosphate buffer (pH 7.8), 0.3 mL of 0.1 mMol EDTA, 0.3 mL of 0.13 Mol methionine, 0.3 mL of 0.75 mMol NBT, 0.3 mL of 0.02 mMol riboflavin, 0.05 mL of enzymatic extract, and 0.25 mL of distilled water. The mixture was exposed to fluorescent light (86.86 μMol/m^2^ s) for 20 min. Then, the solution absorbance was measured at 560 nm in a spectrophotometer NanoDrop™️ 2000 (Thermo Fisher, Waltham, MA, USA). The SOD activity was expressed as U protein/mg of Dry Weight (DW).

#### 4.4.3. CAT Activity Assay

The CAT activity (EC 1.11.1.6) was measured based on the decreasing rate of H_2_O_2_ at 240 nm [54] The reaction was performed by adding 950 μL of 50 mMol potassium phosphate buffer (pH 8.0), 50 μL of enzymatic extract, and 100 μL of 100 mMol H_2_O_2_. The same device was used to measure the absorbance shift at 240 nm for 1 min to determine the decomposition rate of H_2_O_2_ by CAT. The CAT activity was expressed as U protein/mg DW.

#### 4.4.4. PAL Activity

The PAL activity (EC 4.3.1.5) was evaluated by slightly modifying the method described by [8]. L-phenylalanine was used as a substrate, and the released cinnamic acid was quantified based on absorbance at 290 nm. The reaction consisted of adding 230 µL of 0.1 Mol borate buffer (pH 8.8 containing 10 mMol L-phenylalanine) and 20 µL of enzyme extract. The mixture was incubated at 40 °C for 1 h, and the reaction was stopped by adding 50 µL of 1 N HCl. Subsequently, the absorbance of the solution was measured at 290 nm in the NanoDrop spectrophotometer. PAL activity was expressed as U protein/mg DW.

#### 4.4.5. PRO Activity

The Proline (PRO) assay was determined using the method described by [55]. The reaction uses Acid Proline as the standard. The tomato samples (0.045 g) were dissolved in 1.5 mL of sulfosalicylic acid. The mixture was centrifuged at 12,000 rpm for 5 min at 4 °C. Then, 300 μL of the organic phase was separated, and 2 mL of anhidric acid was added and homogenized. The mixture was incubated at 100 °C for 30 min, and the reaction was stopped through thermic shock. In the NanoDrop spectrophotometer, 1 mL of the blended sample was dissolved in 2 mL of sulfosalicylic acid, and its absorbance was read at 520 nm. Proline activity was expressed as μMol PRO/g DW.

### 4.5. Determination of Phenolic Compounds

#### 4.5.1. Extract Preparation

The methanolic extract was prepared by adding 0.2 g of each blended sample to 8 mL of methanol–water solution (50:50) (pH 2) and stirring for one hour. Later, the mixture was centrifuged at 6020 rpm for 10 min at 4 °C; the supernatant was separated and stored. The precipitate was added to 8 mL of acetone–water solution (70:30) and stirred for one hour again. It was centrifuged under the mentioned conditions, and the supernatant was put with that previously recovered. It was stored at −14 °C and protected from light.

#### 4.5.2. Total Polyphenols

Total polyphenols were measured based on the reaction of the Folin–Ciocalteu reagent with sodium carbonate and gallic acid as standards [56]. Briefly, 5 μL of the sample was added to 37.5 μL of Folin–Ciocalteu and mixed with 87.5 µL of sodium carbonate combination and 80 µL of distilled water. Absorbance was measured at 765 nm in a UV-Vis spectrophotometer (BioMate 3, Thermo Scientific). The values were reported in g Gallic Acid Equivalents (GAE)/kg DW (0–0.1).

#### 4.5.3. Flavonoids

Flavonoid content was determined spectrophotometrically according to the method described in [57], using catechin as a standard. First, 5 μL of the methanolic extract was reacted with 7.5 μL of sodium nitrite, 15 μL of aluminum chloride, 172.5 μL of distilled water, and 50 μL of sodium hydroxide. The absorbance was measured in the UV-Vis spectrophotometer at 510 nm [58]. The values obtained were reported in g Catechin Equivalents (Cat Eq)/kg DW (0–0.1).

#### 4.5.4. Total Anthocyanin Content

Anthocyanin quantification was performed with cyanidin-3-glucoside as the standard [59]. First, 175 μL of potassium chloride (0.25 M, pH 1) was reacted with 50 μL of methanolic extract, and the absorbance was measured at 510 and 700 nm. The procedure was repeated with 175 μL of sodium acetate (0.4 M, pH 4.5) and the methanolic extract. Each obtained absorbance value from the UV-Vis spectrophotometer was substituted in Equation (1) to obtain the difference between the potassium chloride’s absorbance, the sodium acetate’s absorbance, and both reactions. After that, the obtained values were replaced in Equation (2) to obtain the total anthocyanin content, represented by g of Cyanidine-3-Glucoside (Cy-3-gly)/kg DW.

(1) $$∆Abs={\left(Abs_{510}-Abs_{700}\right)}_{pH1}-{\left(Abs_{510}-Abs_{700}\right)}_{pH4.5},$$

(2) $$TAcyC=\frac{∆Abs\times MW\times D\times 4.5\times 1000}{\varepsilon },$$

where Δ*Abs* is the difference between pH 1 and 4.5, *TAcyC* is the total anthocyanin content, *M* is a constant of the molecular weight of cyanidin-3-glucoside (445 g), *D* is the number of dilutions, *ε* is the extinction coefficient of cyanidin-3-glucoside (29,600 L/mol cm), and *W* is the sample weight (g).

#### 4.5.5. Carotenoids

Carotenoid quantification was determined spectrophotometrically according to [58]. First, 0.25 g of dry sample was dissolved in a mixture of hexane–acetone–ethanol (2:1:1) for 30 min. Subsequently, 1.5 mL of water was added. The extract was centrifuged at 14,000 rpm for 5 min at 4 °C, and the supernatant was recovered and stored. Afterward, the hexane–acetone–ethanol mixture was added to the precipitate, mixed with water, and centrifuged under the same conditions, and the new supernatant was mixed with the previous one. The absorbance was measured with 5 μL of the extract at 446 nm with the same equipment [60]. The obtained values were substituted in the Lambert–Beer Equation (3) and reported as mg of *β-carotene* (β-Car)/g DW.

(3) $$\beta -carotene=\frac{Abs_{446}M\times DF}{\varepsilon \times D},$$

where *Abs*_446_ is the absorbance reading, *M* is the molecular mass (536.8726 M), *DF* is the Dilution Factor (10.87 g/mol), *ε* is the molar extinction coefficient (24,686/M cm), and D is the distance (1 cm).

### 4.6. Antioxidant Activity

#### 4.6.1. DPPH Inhibition

DPPH radical inhibition was determined using the method reported by [61]. First, 20 μL of methanolic extract was added to 200 μL of the DPPH solution. The absorbance was read at 517 nm using the UV-Vis spectrophotometer. The inhibition percentage was determined by replacing the absorbance values in Equation (4). The Trolox Equivalents Antioxidant Capacity (TEAC) was reported in equivalent mmol of Trolox/kg DW (0–0.8).

(4) $$\%Inhibition=\left(1-\frac{{Abs}_{sample}-{Abs}_{blank}}{{Abs}_{control}\;}\right)\times 100$$

where *Abs_sample_* is the sample reading, *Abs_blank_* corresponds to a water and methanol hint, and *Abs_control_* is the reference of the methanol and DPPH solution.

#### 4.6.2. ABTS Inhibition

The ABTS assay was determined using the method reported by [60]. The ABTS solution was prepared with potassium persulfate after incubation for 12 h and protected from light. The samples consisted of 20 μL of the methanolic extract with 230 μL of ABTS solution, and their absorbance was read at 734 nm with the UV-Vis spectrophotometer. The obtained absorbance values were substituted in Equation (4) to calculate the inhibition percentage. The TEAC was reported to be equivalent to mmol of Trolox/kg DW (0–0.6).

### 4.7. Statistical Analysis

The fruits of the three varieties were analyzed in triplicate with an experimental unit of 6 samples for treatment. The treatments were compared using a one-way ANOVA, followed by Tukey’s test for mean comparison. The results were reported as mean ± standard deviations. Significant differences among the varieties were determined through variance analysis and Tukey’s tests (*p* < 0.05) using Statgraphics^®^ Centurion XVI statistical software (StatPoint Technologies Inc., Bedford, MA, USA, 2010).

Additionally, an analysis of the interaction between enzymatic activities and non-enzymatic compounds was performed using a correlation analysis to evaluate the relationships between SOD, CAT, PRO, PAL, total phenolic acids, total flavonoids, anthocyanins, and β-carotenes. Pearson’s correlation coefficient was used to measure the strength and direction of the linear relationships between variables.

## 5. Conclusions

The fruit analysis of the Kumato variety had the best results of the three analyzed varieties. Kumato significantly differed in the total phenolic, flavonoid, and β-carotene content. It also presented greater antioxidant activity in DPPH and ABTS. In addition, it presented significant differences in CAT and PAL enzymatic activities. In comparison, the Indigo Rose variety had a greater significant difference in anthocyanins and SOD. The Indigo Cherry Drops variety accumulated PRO. Further studies are required to clarify the health benefits of these fruits. This research may motivate the production of black cherry tomatoes. Its curious color and nutritional contribution may attract different applications in gastronomy and not only for fresh consumption. Promoting the nutraceutical benefits and the potential reduction effects of ROS for a healthier life is important for final consumer acceptance of cherry tomatoes.

## Figures and Tables

**Table 1:** DPPH and ABTS radical inhibition rate and quantification of Trolox Equivalent Antioxidant Capacity (TEAC) of each black cherry tomato variety.

Black Cherry Tomato Variety	ABTS		DPPH	
	% Inhibition	TEAC (Trolox/kg DW)	% Inhibition	TEAC (Trolox/kg DW)
Indigo Cherry Drops	58.71 ± 0.32 ^b^	117.98 ± 1.68 ^b^	47.48 ± 3.75 ^b^	358.23 ± 31.67 ^b^
Indigo Rose	41.39 ± 0.50 ^c^	27.58 ± 2.62 ^c^	65.83 ± 6.83 ^c^	513.13 ± 57.60 ^c^
Kumato	74.73 ± 0.34 ^a^	201.59 ± 1.78 ^a^	87.06 ± 3.41 ^a^	692.23 ± 28.74 ^a^

Values are presented as mean ± standard deviation. Values with the different letters are statistically different according to Tukey’s test (α = 0.05). TEAC means mMol of TEAC.

**Table 2:** Composition of the Steiner nutrient solution for each phenological stage of black cherry tomato cultivation (the total volume of the solution was 5000 L) [52].

Compound	Seed–Seedling (kg)	Plant Growth (kg)	Fruit Growth (kg)
Phosphoric acid	0.470	0.470	-
Nitric acid	-	-	0.604
Calcium nitrate	6.500	2.950	3.850
Sulphuric acid	-	-	0.732
Potassium sulphate	1.875	1.000	-
Potassium nitrate	2.500	-	2.900
Magnesium nitrate	-	0.650	0.800
Magnesium sulphate	3.125	0.800	1.300
Copper sulphate	0.050	-	-
Potassium phosphate	-	0.820	-
Potassium mono phosphate	-	-	1.100
Potassium chloride	-	0.100	1.200
Iron	0.125	0.076	0.075
Manganese	0.025	0.030	0.030
Copper	-	0.003	0.002
Zinc	0.025	0.011	0.010
Boron	0.025	0.010	0.010
Molybdenum	-	0.001	0.001

## Data Availability

The original contributions presented in this study are included in the article. Further inquiries can be directed to the corresponding author(s).
